# Nutrient intake and diet quality in individuals with hyperuricemia: a matched population study

**DOI:** 10.3389/fnut.2026.1719104

**Published:** 2026-02-04

**Authors:** Ehn-Young Kim, Seok-Jae Heo, Yaeji Lee, Tae-Hwa Han, Yu-Jin Kwon, Ji-Won Lee

**Affiliations:** 1 Department of Family Medicine, Severance Hospital, Yonsei University College of Medicine, Seoul, Republic of Korea; 2 Biostatistics Collaboration Unit, Department of Biomedical Systems Informatics, Yonsei University College of Medicine, Seoul, Republic of Korea; 3 Division of Biostatistics, Department of Biomedical Systems Informatics, Yonsei University College of Medicine, Seoul, Republic of Korea; 4 Cerebro-Cardiovascular Diseases Research Center (Health-IT), Yonsei University College of Medicine, Severance Hospital, Seoul, Republic of Korea; 5 Department of Family Medicine, Yongin Severance Hospital, Yonsei University College of Medicine, Yongin-si, Gyeonggi-do, Republic of Korea; 6 Institute for Innovation in Digital Healthcare, Yonsei University, Seoul, Republic of Korea

**Keywords:** dietary intake, Healthy Eating Index, hyperuricemia, nutrient intake, uric acid

## Abstract

**Background:**

Dietary factors influence hyperuricemia; however, comprehensive evaluations integrating nutrient intake, dietary patterns, and overall diet quality remain limited. This study aimed to address this gap by assessing nutrient intake using the Korean Dietary Reference Intakes (KDRIs) and diet quality using the Korean Healthy Eating Index (KHEI).

**Methods:**

In this cross-sectional study using Korea National Health and Nutrition Examination Survey (KNHANES) data, 24,026 Korean adults were eligible prior to matching, stratified by sex, and then classified according to hyperuricemia status within each sex. After 1:1 propensity score matching based on age and body mass index, 10,268 participants were included in the final analysis. Dietary intake levels were assessed using a 24-h dietary recall, and nutrient intake and dietary quality were evaluated using the KDRIs and KHEI, respectively.

**Results:**

The KDRI-based analysis showed that a lower proportion of individuals with hyperuricemia met or exceeded the recommended carbohydrate intake in both sexes (men: 39.1% vs. 34.0%, *p* < 0.001; women: 54.0% vs. 50.1%, *p* < 0.001), whereas a higher proportion of women with hyperuricemia had protein intake exceeding the recommended intake levels (10.6% vs. 8.1%, *p* = 0.003). In the hyperuricemia group, the proportion of participants meeting the KDRIs for fiber and minerals, such as magnesium and zinc, was significantly lower in both sexes. Among men, a lower proportion met the KDRI criteria for calcium, iron, potassium, folate, and vitamins B1, B2, and C intake. The mean KHEI score was significantly lower in the hyperuricemia group than in the control group, in both sexes. Among men, the proportion of individuals with component scores ≥7 for whole grains, total fruit, fresh fruit, total vegetables, and breakfast was significantly lower in the hyperuricemia group (all *p* < 0.01). Among women, the hyperuricemia group consumed a lower proportion of total vegetables (*p* = 0.003).

**Conclusion:**

This study highlights the importance of a comprehensive dietary approach in managing hyperuricemia. These findings support the implementation of individualized strategies focused on balanced macronutrient intake, quality and sources of macronutrients, increased consumption of fiber-rich plant-based foods, adequate micronutrient intake, and healthy eating practices.

## 1 Introduction

Hyperuricemia, defined as an elevated serum uric acid (SUA) concentration, is not only a significant risk factor for gout but also an important metabolic risk factor associated with hypertension (HTN), chronic kidney disease, insulin resistance, type 2 diabetes mellitus (T2DM), cardiovascular disease, and metabolic syndrome (1–4). Uric acid (UA), the final product of purine metabolism, is primarily produced through the hepatic degradation of endogenous purines, with a smaller proportion originating from dietary purine intake (3). The balance between hepatic urate synthesis and elimination through renal and intestinal excretion pathways regulates the serum urate levels (3). Genetic predispositions, metabolic conditions, and environmental factors, particularly dietary habits, influence this equilibrium (3, 5).

Diet plays a crucial role in UA metabolism and remains a clinically meaningful and modifiable intervention target due to its potential for external regulation (3, 4). Hyperuricemia is associated with high consumption of red meat, shellfish, alcohol, and sugar-sweetened beverages, as well as a low intake of vegetables, dietary fiber, folate, and micronutrient-rich foods (3, 4, 6–8). Adherence to plant-based or anti-inflammatory nutritional patterns, including the Dietary Approaches to Stop Hypertension and Mediterranean diets, reduces SUA levels and lowers the risk of hyperuricemia and gout (9, 10).

However, evidence regarding the association between diet and hyperuricemia remains inconsistent, with some studies reporting nonsignificant or even conflicting outcomes depending on factors such as the study population, dietary assessment methods, and dietary context (11, 12). Korean dietary patterns differ markedly from those of Western populations and have undergone rapid nutritional transitions in recent decades. Although the association between diet and hyperuricemia in Asian populations has been investigated in several studies, evidence based on nationally representative data from South Korea is limited. Moreover, despite the growing recognition of hyperuricemia as a significant metabolic risk factor, nutrient adequacy in relation to standardized guidelines, such as the Dietary Reference Intakes (DRIs), and other qualitative aspects of diet have been overlooked in previous research.

This study aimed to identify dietary factors associated with hyperuricemia in the Korean population using data from the nationally representative Korea National Health and Nutrition Examination Survey (KNHANES) and to perform 1:1 propensity score matching (PSM) between individuals with hyperuricemia and controls to minimize potential confounding factors. The intake levels of various nutrients were evaluated based on the Korean Dietary Reference Intakes (KDRIs) values, dietary habits, and diet quality using the Korean Healthy Eating Index (KHEI) to provide an integrated perspective on the nutritional factors associated with hyperuricemia in the Korean population.

## 2 Materials and methods

### 2.1 Study population

This cross-sectional analysis was based on data from the KNHANES administered by the Korea Centers for Disease Control and Prevention under the National Health Promotion Act. The KNHANES is a nationally representative survey conducted annually since 1998, aiming to collect comprehensive information on nutritional intake, health-related behaviors, and the prevalence of chronic diseases in the Korean population using a stratified, multistage probability sampling method. The survey includes components such as a health interview, physical examination, and nutritional assessment of households.

**Figure 1:**
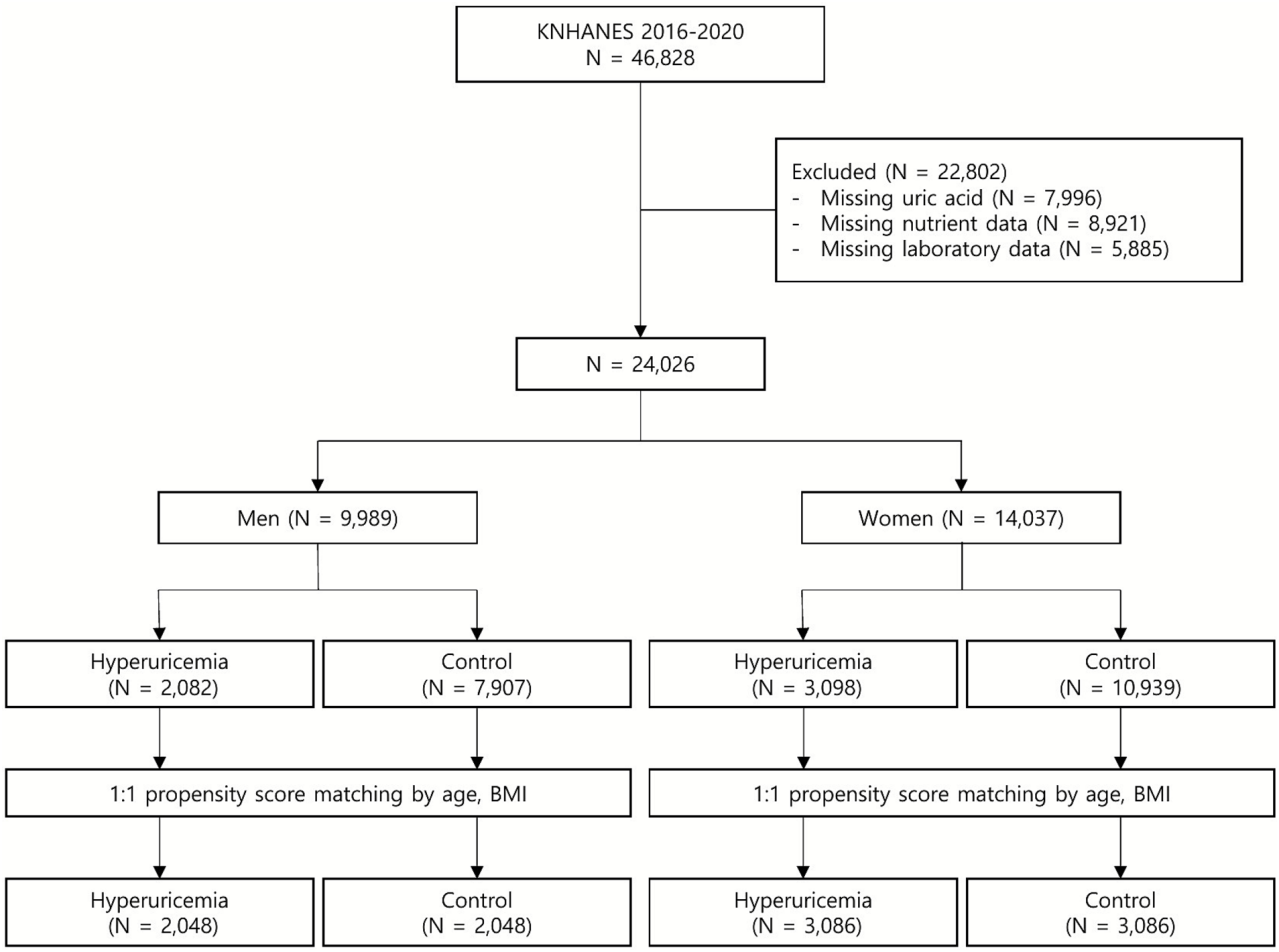
Flow chart of the study population selection process.

A flowchart illustrating participant inclusion in the study from the overall KNHANES population is shown in Figure 1. Data from 46,828 participants enrolled in the KNHANES between 2016 and 2021 were obtained for this study. Participants were excluded if they had incomplete information on nutrient intake (*n* = 8,921), SUA levels (*n* = 7,996), or other laboratory parameters (*n* = 5,885). Ultimately, 24,026 participants (19–80 years of age) were eligible for analysis before PSM and stratified by sex and categorized into hyperuricemia and control groups. After performing PSM based on age and body mass index (BMI), 10,268 participants were included in the final analysis: 2,048 men with hyperuricemia, 2,048 men without hyperuricemia, 3,086 women with hyperuricemia, and 3,086 women without hyperuricemia. Informed consent was obtained from all participants before the survey. This study was conducted in accordance with the principles of the 1964 Declaration of Helsinki. The Institutional Review Board of Severance Hospital, Yonsei University Health System, approved this study (IRB No. 4-2025-1068). This study was conducted and reported in accordance with the Strengthening the Reporting of Observational Studies in Epidemiology (STROBE) guidelines (13).

### 2.2 Definition of hyperuricemia

The hyperuricemia threshold was defined based on the sex-specific 20th percentile of SUA levels among the study participants. Accordingly, the cut-off values were set at 7 mg/dL for men and 5.7 mg/dL for women. These threshold values were consistent with the primary cutoff values defined in a previous study that analyzed the prevalence of gout, hyperuricemia, and decadal trends using data from the United States National Health and Nutrition Examination Survey (2007–2016), where the prevalence of hyperuricemia was 20.2 and 20.0% in men and women, respectively (14).

### 2.3 Measurements and variables

All participants underwent physical and laboratory examinations according to the standard procedures. Self-administered questionnaires were used to collect medical and lifestyle data. Dietary intake was evaluated using the 24-h recall method. Height was measured to the first decimal place (0.1 cm) using a stadiometer, and weight was measured to the first decimal place (0.1 kg) using an electronic balance. Waist circumference (WC) was measured at the midpoint between the inferior margin of the rib cage and the iliac crest. BMI (kg/m^2^) was calculated as weight in kilograms divided by height in meters squared. Blood pressure in the right arm was measured using standard mercury sphygmomanometer after the participant rested in a sitting position for 5 min. The mean blood pressure (MBP) was calculated as follows: diastolic BP (DBP) + 1/3 × (systolic BP [SBP] − DBP).

Trained interviewers collected lifestyle data using questionnaires. Alcohol intake was defined as alcohol consumption at least once a month during the previous year. Smoking status was defined as having smoked >100 cigarettes in their lifetime and currently being a smoker. Physical activity was defined as engaging in at least 2 h and 30 min of moderate-intensity physical activity per week, 1 h and 15 min of vigorous-intensity physical activity per week, or a combination of both (where 1 min of vigorous-intensity activity was considered equivalent to 2 min of moderate-intensity activity). Trained medical staff collected blood samples from each participant’s antecubital vein after an 8 h fasting period. SUA levels were measured using the colorimetric method with a Hitachi Automatic Analyzer 7600/7600-210.

Chronic diseases were defined as follows: HTN was characterized by an SBP ≥ 140 mmHg, DBP ≥ 90 mmHg, or current use of antihypertensive medications. T2DM was defined as a fasting plasma glucose (FPG) level ≥126 mg/dL, hemoglobin A1c (HbA1c) ≥ 6.5%, a self-reported diagnosis of T2DM, or use of anti-diabetic agents. Dyslipidemia was defined as a total cholesterol (TC) level ≥ 240 mg/dL, a triglyceride (TG) level ≥ 200 mg/dL, or the use of lipid-lowering medications. Metabolic syndrome was defined as three or more of the following criteria: (i) abdominal obesity; (ii) SBP ≥ 130 mmHg, DBP ≥ 85 mmHg, or treatment with anti-hypertensive therapy; (iii) FPG ≥ 100 mg/dL or treatment with anti-diabetic agents or insulin; (iv) TG ≥ 150 mg/dL or use of lipid-lowering drugs; and (v) high-density lipoprotein cholesterol <50 mg/dL in women or <40 mg/dL in men (15).

### 2.4 Nutrient intake assessment and reference data

Dietary information was collected using the 24-h dietary recall method. Trained interviewers conducted computer-assisted personal interviews to assess the food intake of each study participant over the past 24 h, collecting detailed nutritional information, including time, location, food type, quantity, and cooking method. KNHANES uses the most up-to-date nutrient composition database available for each survey year to calculate nutrient intake. Nutritional information on individual dietary items was obtained from the Food Composition Table published by the National Institute of Agricultural Sciences. The intake levels of 18 nutrients were classified as either above or below the corresponding KDRI. The proportion of participants with intake levels above or below the KDRIs was then compared between the hyperuricemia and control groups.

DRIs are a set of reference values used to assess and plan nutrient intake in healthy populations. In this study, we established nutritional intake criteria based on the 2020 Korean Dietary Reference Intakes (Ministry of Health & Welfare’s research project). Macronutrient intake was assessed considering the acceptable macronutrient distribution range (AMDR), defined as the recommended range of energy intake for each macronutrient. The KDRI AMDRs were 55–65% for carbohydrates, 7–20% for proteins, and 15–30% for fats (16). For this study, the upper limit of each AMDR was used as the reference intake criterion. For nutrients, such as potassium, sodium, dietary fiber, and vitamin E, adequate intake values were applied, defined as the recommended average daily intake level based on observed or experimentally derived estimates in healthy populations (17). The DRI for cholesterol was determined based on chronic disease risk reduction values, as cholesterol recommendations are not included in the 2020 KDRIs. Therefore, reference values established by U.S. and Canadian guidelines were used (18). The DRIs of other nutrients were set based on the recommended nutrient intake, which is the average daily nutrient intake sufficient to meet the nutrient requirements of approximately 97–98% of all healthy individuals. The detailed criteria for the sex- and age-specific DRIs used in this study are presented in Supplementary Table S1.

### 2.5 Dietary habit and quality assessments

Weekly dietary intake was analyzed using data from a semi-quantitative Food Frequency Questionnaire (FFQ) comprising 112 items. The FFQ is a valuable tool for examining the relationship between diet and chronic diseases in large population-based studies. The development process of the FFQ has been described previously (19). In the KNHANES 2016–2021, FFQ data were limited to 2016. The 112 items were categorized into 18 food groups and three types of alcoholic beverages for analysis.

KHEI is a dietary quality index developed based on the Dietary Guidelines for Americans (20, 21). It is used to evaluate adherence to nutritional recommendations and comprises 14 components. The total KHEI score ranged from 0 to 100, with higher scores indicating better overall dietary quality. The 14 components included eight adequacy items, representing recommended food groups for sufficient intake (i.e., having breakfast; whole grains; fruits excluding fruit juice; fruits including fruit juice; total vegetables including kimchi and pickles; total vegetables excluding kimchi and pickles; meat, fish, eggs, and legumes; and milk and dairy products), and six moderation items (i.e., saturated fatty acids, sodium, empty-calorie foods such as sweet and beverages, carbohydrates, total fat, and total energy intake). The adequacy components reflect food groups encouraged for regular consumption, with higher scores representing higher intake. In contrast, the moderation components are those for which a lower intake is recommended; thus, higher scores indicate a lower and more desirable intake. Each component contributed a maximum of 5–10 points, yielding a total possible score of 100 points. Further details regarding KHEI scoring have been described in previous studies (20, 21). Each component score was categorized into three groups: low (0–4), middle (4–7), and high (≥7). For components with a maximum of 5 points, the scores were doubled to apply the same classification thresholds.

### 2.6 Statistical analysis

Given the well-documented biological and behavioral differences in serum uric acid metabolism between men and women (22–24), all analyses were conducted separately by sex. This sex-stratified analytical approach was adopted to account for potential effect modification by sex and to minimize dilution of sex-specific associations that may occur in pooled analyses.

Within each sex, 1:1 PSM was performed between participants with hyperuricemia and matched controls to reduce potential confounding and improve group comparability. Matching was based on age and BMI, which are key determinants of hyperuricemia and diet-related characteristics. Other lifestyle-related covariates were not included in the matching process but were adjusted for in subsequent regression analyses to avoid excessive loss of sample size and over-restriction of the matched sample. Balance in baseline characteristics between the matched groups was assessed using standardized mean differences (SMDs), and age and BMI achieved adequate balance after matching (SMD < 0.1).

The baseline characteristics are summarized with descriptive statistics, with continuous variables presented as means with standard deviations and categorical variables as frequencies and percentages. To account for the within-pair correlation inherent in the matched design, the matched hyperuricemia and control groups were compared using generalized estimating equations (25, 26), with additional adjustments for alcohol consumption, smoking status, physical activity, and total energy intake. Multicollinearity among covariates included in the adjusted models was assessed using variance inflation factors (VIFs), and no evidence of problematic multicollinearity was observed (all VIFs < 1.3). For sensitivity analysis, additional models were fitted with further adjustment for serum creatinine to evaluate the potential influence of kidney function on the observed associations.

The primary aim of this study was to examine associations and comparative dietary characteristics rather than generate nationally representative estimates, and the propensity score-matched analytic sample no longer preserved the original complex sampling structure of KNHANES; therefore, survey weights and complex sampling design variables were not applied. All statistical analyses were performed using the R software (version 4.3.2; R Foundation for Statistical Computing, Vienna, Austria).^1^ All statistical analyses were conducted using two-sided tests, with statistical significance defined as *p* < 0.05.

## 3 Results

### 3.1 Clinical and nutritional characteristics of the propensity score–matched population by hyperuricemia status

The clinical characteristics of the matched population, categorized by hyperuricemia status, are presented in Table 1. The mean age and BMI were 48.6 ± 17.1 years and 25.8 ± 3.6 kg/m^2^ in men, and 54.7 ± 16.4 years and 25.2 ± 3.9 kg/m^2^ in women. Individuals with hyperuricemia had higher WC and MBP and a higher prevalence of HTN, dyslipidemia, and metabolic syndrome than the controls in both sexes. Serum TC, TG, low-density lipoprotein cholesterol, and creatinine levels were significantly higher in men and women with hyperuricemia. The proportion of individuals reporting alcohol consumption was significantly higher in the hyperuricemia group than in the control group. The hyperuricemia group had lower carbohydrate intake in both sexes. In contrast, the intake of protein, total fat, and saturated fatty acids was significantly higher in women with hyperuricemia. The hyperuricemia group had a significantly lower intake of minerals, including magnesium, folate, and dietary fiber, than the control group in both sexes. The KHEI score was also significantly lower in the hyperuricemia group than in the control group for both sexes.

**Table 1:** Characteristics of the 1:1 propensity score–matched study population.

Variable	Men			Women		
	Controls (*N* = 2,048)	Hyperuricemia (*N* = 2,048)	*p*-value	Controls (*N* = 3,086)	Hyperuricemia (*N* = 3,086)	*p*-value
Age (y)	48.9 ± 17.0	48.4 ± 17.3	–	54.9 ± 15.4	54.5 ± 17.4	–
BMI (kg/m^2^)	25.8 ± 3.7	25.7 ± 3.5	–	25.1 ± 3.9	25.1 ± 3.9	–
WC (cm)	90.1 ± 9.7	90.3 ± 9.1	**0.002**	84.0 ± 10.2	84.4 ± 10.1	**<0.001**
MBP (mmHg)	92.5 ± 10.0	93.9 ± 11.0	**<0.001**	89.8 ± 11.0	90.3 ± 11.1	**0.035**
Alcohol intake (yes)	1,421 (69.4%)	1,491 (72.8%)	**0.010**	1,140 (36.9%)	1,199 (38.9%)	0.421
Smoking (yes)	621 (30.3%)	654 (31.9%)	0.518	122 (4.0%)	192 (6.2%)	**<0.001**
Physical activity (yes)	1,011 (49.4%)	973 (47.5%)	0.150	1,179 (38.2%)	1,189 (38.5%)	0.843
Hypertension (yes)	727 (35.5%)	816 (39.8%)	**<0.001**	1,125 (36.5%)	1,293 (41.9%)	**<0.001**
Diabetes (yes)	367 (17.9%)	252 (12.3%)	**<0.001**	490 (15.9%)	541 (17.5%)	0.153
Dyslipidemia (yes)	759 (37.1%)	940 (45.9%)	**<0.001**	1,189 (38.5%)	1,349 (43.7%)	**<0.001**
Metabolic syndrome (yes)	707 (34.5%)	851 (41.6%)	**<0.001**	1,065 (34.5%)	1,371 (44.4%)	**<0.001**
Glucose (mg/dL)	105.5 ± 27.5	101.9 ± 19.3	**<0.001**	101.7 ± 23.6	102.6 ± 22.8	0.137
TC (mg/dL)	189.8 ± 38.8	198.2 ± 40.3	**<0.001**	193.4 ± 39.8	197.1 ± 41.8	**<0.001**
TG (mg/dL)	149.0 ± 112.0	191.7 ± 159.6	**<0.001**	115.1 ± 69.6	139.3 ± 96.0	**<0.001**
HDL-C (mg/dL)	47.7 ± 11.3	45.0 ± 10.3	**<0.001**	53.9 ± 12.4	51.5 ± 12.4	**<0.001**
LDL-C (mg/dL)	114.6 ± 34.2	119.5 ± 35.9	**<0.001**	116.9 ± 35.3	119.0 ± 37.5	**0.016**
Uric acid (mg/dL)	5.5 ± 0.9	7.8 ± 0.8	**<0.001**	4.1 ± 0.7	5.9 ± 0.8	**<0.001**
Creatinine	0.9 ± 0.2	1.0 ± 0.3	**<0.001**	0.7 ± 0.2	0.8 ± 0.3	**<0.001**
HEI	60.6 ± 13.6	58.3 ± 13.2	**<0.001**	63.9 ± 13.1	62.8 ± 13.6	**0.039**
Total energy intake, kcal/day	2,277.6 ± 969.2	2,238.2 ± 952.9	**<0.001**	1,604.8 ± 655.4	1,569.1 ± 666.2	**<0.001**
Carbohydrate (%)	59.1 ± 14.3	57.4 ± 15.1	**<0.001**	65.2 ± 12.6	63.7 ± 13.7	**<0.001**
Protein (%)	14.9 ± 4.4	14.7 ± 4.8	0.167	14.3 ± 4.1	14.6 ± 4.5	**0.019**
Fat (%)	20.5 ± 9.4	20.7 ± 9.9	0.291	19.2 ± 9.4	19.8 ± 9.9	**0.008**
SFA (%)	6.4 ± 3.6	6.6 ± 3.7	0.123	6.0 ± 3.6	6.3 ± 3.9	**0.004**
MUFA (%)	6.6 ± 3.8	6.7 ± 4.0	0.141	6.0 ± 3.7	6.2 ± 3.8	0.067
PUFA (%)	5.4 ± 2.9	5.3 ± 3.0	0.330	5.3 ± 2.8	5.3 ± 3.0	0.375
PUFA / SFA	1.1 ± 0.7	1.0 ± 0.6	**0.013**	1.1 ± 0.7	1.1 ± 0.8	0.209
PUFA + MUFA /SFA	15.2 ± 10.6	14.6 ± 10.2	0.209	10.7 ± 7.4	10.6 ± 7.7	0.238
Omega-3 fatty acid, g/day	2.1 ± 2.0	2.0 ± 1.9	0.208	1.7 ± 2.2	1.63 ± 1.84	0.960
Omega-6 fatty acid, g/day	11.9 ± 9.2	11.5 ± 8.8	0.277	8.0 ± 6.3	8.0 ± 6.5	0.169
Cholesterol, mg/day	291.6 ± 250.8	279.5 ± 240.3	0.261	188.7 ± 171.5	192.5 ± 188.5	0.060
Fiber, g/day	28.3 ± 14.5	26.4 ± 13.8	**<0.001**	24.5 ± 13.6	23.2 ± 13.4	**0.005**
Mg, mg/day	358.5 ± 160.8	335.7 ± 151.9	**<0.001**	284.1 ± 142.5	270.9 ± 134.0	**0.004**
Ca, mg/day	558.7 ± 307.9	522.6 ± 293.6	**<0.001**	455.3 ± 296.2	437.5 ± 274.2	0.123
Iron, mg/day	12.7 ± 8.3	11.9 ± 7.5	**0.002**	9.6 ± 5.7	9.1 ± 5.7	0.067
Na, mg/day	3,998.6 ± 2,184.2	3,857.4 ± 2,178.8	0.112	2,806.6 ± 1,885.4	2,644.0 ± 1,675.5	**0.003**
K, mg/day	3,123.9 ± 1,395.7	2,962.7 ± 1,351.3	**<0.001**	2,558.1 ± 1,296.8	2,459.1 ± 1,274.7	0.070
Zinc, mg/day	12.1 ± 6.2	11.6 ± 6.0	**0.032**	8.8 ± 4.2	8.6 ± 4.3	0.657
Vitamin A, μg RAE	425.1 ± 538.9	402.3 ± 393.4	0.297	350.8 ± 364.8	342.9 ± 375.9	0.971
Vitamin E, mg/day	7.8 ± 4.6	7.5 ± 4.4	**0.020**	5.8 ± 3.5	5.6 ± 3.6	0.791
Vitamin B1, mg/day	1.5 ± 0.9	1.4 ± 0.9	0.198	1.1 ± 0.6	1.0 ± 0.6	0.106
Vitamin B2, mg/day	1.82 ± 1.04	1.8 ± 1.0	0.114	1.3 ± 0.7	1.3 ± 0.8	0.917
Niacin, mg/day	15.3 ± 9.2	14.9 ± 9.1	0.633	10.6 ± 5.9	10.7 ± 6.5	0.061
Vitamin C, mg/day	69.9 ± 107.1	64.2 ± 106.7	0.154	62.8 ± 72.9	59.5 ± 72.6	0.324
Folate, μg/day	361.4 ± 183.3	330.6 ± 165.6	**<0.001**	298.3 ± 154.8	283.8 ± 156.5	**0.009**

BMI, body mass index; WC, waist circumference; MBP, mean blood pressure; TC, total cholesterol; TG, triglycerides; HDL-C, high-density lipoprotein cholesterol; LDL-C, low-density lipoprotein cholesterol; HEI, healthy eating index; SFA, saturated fatty acids; MUFA, monounsaturated fatty acids; PUFA, polyunsaturated fatty acids; Mg, magnesium; Ca, calcium; Na, sodium; K, potassium; RAE, retinol activity equivalents.

Data are presented as mean ± standard deviation for continuous variables or as number (%) for categorical variables.

Age and BMI were matched in a 1:1 ratio using propensity score matching. *p*-values were calculated using generalized estimating equations with additional adjustments for alcohol consumption, smoking status, physical activity, and total energy intake. All *p*-values are two-sided. Statistical significance is defined as *p* < 0.05, and statistically significant results are highlighted in bold.

### 3.2 Nutrient intake according to the KDRIs in hyperuricemia and control groups

The daily nutrient intake levels relative to the KDRIs for the hyperuricemia and control groups, stratified by sex, are shown in Table 2. Among both men and women, the proportion of individuals with carbohydrate intake (%) above the KDRI was significantly lower in the hyperuricemia group than in the control group (men: 39.1% vs. 34.0%, *p* < 0.001; women: 54.0% vs. 50.1%, *p* < 0.001). Among women, the proportion exceeding the KDRI for protein intake (%) was significantly higher in the hyperuricemia group (10.6% vs. 8.1%, *p* = 0.003). Although the proportion of individuals with fat intake (%) above the KDRI was higher in the hyperuricemia group in both sexes, this difference was not statistically significant. Regarding fiber and minerals, such as magnesium, zinc, and sodium, the proportion meeting or exceeding the KDRIs was lower in the hyperuricemia group for both sexes. In men, the proportions meeting or exceeding the KDRIs for calcium, iron, potassium, folate, and vitamins B1, B2, and C were also significantly lower in the hyperuricemia group than in the control group. Overall, these findings indicate that individuals with hyperuricemia tend to adhere poorly to the recommended intake levels for carbohydrates, dietary fiber, and several key micronutrients.

**Table 2:** Comparison of daily nutrient intake between individuals with hyperuricemia and controls in the 1:1 propensity score–matched study population.

Nutrients	Men					Women				
	Controls (*N* = 2,048)		Hyperuricemia (*N* = 2,048)		*p*-value	Controls (*N* = 3,086)		Hyperuricemia (*N* = 3,086)		*p*-value
	Above DRI group, n (%)	Below DRI group, *n* (%)	Above DRI group, *n* (%)	Below DRI group, *n* (%)		Above DRI group, *n* (%)	Below DRI group, *n* (%)	Above DRI group, *n* (%)	Below DRI group, *n* (%)	
Carbohydrate (%)	801 (39.1%)	1,247 (60.9%)	696 (34.0%)	1,352 (66.0%)	**<0.001**	1,667 (54.0%)	1,419 (46.0%)	1,545 (50.1%)	1,541 (49.9%)	**<0.001**
Protein (%)	226 (11.0%)	1,822 (89.0%)	220 (10.7%)	1,828 (89.3%)	0.647	251 (8.1%)	2,835 (91.9%)	327 (10.6%)	2,759 (89.4%)	**0.003**
Fat (%)	301 (14.7%)	1,747 (85.3%)	343 (16.7%)	1,705 (83.3%)	0.064	414 (13.4%)	2,672 (86.6%)	463 (15.0%)	2,623 (85.0%)	0.146
Cholesterol, mg/day	788 (38.5%)	1,260 (61.5%)	752 (36.7%)	1,296 (63.3%)	0.313	686 (22.2%)	2,400 (77.8%)	688 (22.3%)	2,398 (77.7%)	0.737
Fiber, g/day	838 (40.9%)	1,210 (59.1%)	735 (35.9%)	1,313 (64.1%)	**0.016**	1,750 (56.7%)	1,336 (43.3%)	1,585 (51.4%)	1,501 (48.6%)	**0.002**
Mg, mg/day	831 (40.6%)	1,217 (59.4%)	719 (35.1%)	1,329 (64.9%)	**0.002**	1,387 (44.9%)	1,699 (55.1%)	1,233 (40.0%)	1,853 (60.0%)	**0.003**
Ca, mg/day	408 (19.9%)	1,640 (80.1%)	327 (16.0%)	1,721 (84.0%)	**0.005**	340 (11.0%)	2,746 (89.0%)	329 (10.7%)	2,757 (89.3%)	0.860
Iron, mg/day	1,218 (59.5%)	830 (40.5%)	1,100 (53.7%)	948 (46.3%)	**0.001**	1,252 (40.6%)	1,834 (59.4%)	1,185 (38.4%)	1,901 (61.6%)	0.378
Na, mg/day	1,922 (93.8%)	126 (6.2%)	1,874 (91.5%)	174 (8.5%)	**0.012**	2,509 (81.3%)	577 (18.7%)	2,445 (79.2%)	641 (20.8%)	**0.041**
K, mg/day	689 (33.6%)	1,359 (66.4%)	609 (29.7%)	1,439 (70.3%)	**0.038**	570 (18.5%)	2,516 (81.5%)	534 (17.3%)	2,552 (82.7%)	0.683
Zinc, mg/day	1,240 (60.5%)	808 (39.5%)	1,148 (56.1%)	900 (43.9%)	**0.028**	1,736 (56.3%)	1,350 (43.7%)	1,594 (51.7%)	1,492 (48.3%)	**0.024**
Vitamin A, μg RAE	190 (9.3%)	1,858 (90.7%)	207 (10.1%)	1,841 (89.9%)	0.137	381 (12.3%)	2,705 (87.7%)	375 (12.2%)	2,711 (87.8%)	0.794
Vitamin E, mg/day	271 (13.2%)	1,777 (86.8%)	253 (12.4%)	1,795 (87.6%)	0.702	157 (5.1%)	2,929 (94.9%)	158 (5.1%)	2,928 (94.9%)	0.827
Vitamin B1, mg/day	1,152 (56.3%)	896 (43.8%)	1,071 (52.3%)	977 (47.7%)	**0.041**	1,286 (41.7%)	1,800 (58.3%)	1,228 (39.8%)	1,858 (60.2%)	0.877
Vitamin B2, mg/day	1,191 (58.2%)	857 (41.8%)	1,113 (54.3%)	935 (45.7%)	**0.040**	1,621 (52.5%)	1,465 (47.5%)	1,537 (49.8%)	1,549 (50.2%)	0.508
Niacin, mg/day	785 (38.3%)	1,263 (61.7%)	732 (35.7%)	1,316 (64.3%)	0.170	690 (22.4%)	2,396 (77.6%)	721 (23.4%)	2,365 (76.6%)	0.084
Vitamin C, mg/day	360 (17.6%)	1,688 (82.4%)	302 (14.7%)	1,746 (85.3%)	**0.044**	498 (16.1%)	2,588 (83.9%)	501 (16.2%)	2,585 (83.8%)	0.497
Folate, μg/day	705 (34.4%)	1,343 (65.6%)	591 (28.9%)	1,457 (71.1%)	**0.001**	630 (20.4%)	2,456 (79.6%)	600 (19.4%)	2,486 (80.6%)	0.883

DRI, dietary reference intake; Mg, magnesium; Ca, calcium; Na, sodium; K, potassium; RAE, retinol activity equivalent. Reference values for nutrients were based on the following criteria according to sex and age:

Acceptable macronutrient distribution ranges (AMDR): carbohydrate (%), protein (%), and fat (%).

Recommended nutrient intake: Mg, Ca, iron, vitamins A, B1, B2, and C, niacin, zinc, and folate.

Adequate intake: fiber, Na, K, and vitamin E.

Cholesterol levels were set based on chronic disease endpoints.

Age and BMI were matched in a 1:1 ratio using propensity score matching. *p*-values were calculated using generalized estimating equations with additional adjustments for alcohol consumption, smoking status, physical activity, and total energy intake. All *p*-values are two-sided. Statistical significance is defined as *p* < 0.05, and statistically significant results are highlighted in bold.

### 3.3 Weekly dietary intake and alcohol consumption in hyperuricemia and control groups

A comparison of the weekly dietary intake and alcohol consumption between the hyperuricemia and control groups is shown in Table 3. In the weekly dietary intake analysis, women in the hyperuricemia group consumed fewer whole grains and more refined grains than those in the control group. The weekly intake of other non-fish seafood and shellfish was significantly higher in women with hyperuricemia than that in the controls (*p* = 0.045). Regarding alcohol consumption, the hyperuricemia group demonstrated a significantly higher intake of soju (Korean distilled liquor) than the control group in both sexes (*p* = 0.040 for men and *p* = 0.012 for women). Weekly beer intake was also higher in the hyperuricemia group for both sexes; however, the difference was only statistically significant among women (*p* = 0.035).

**Table 3:** Comparison of weekly dietary intake and alcohol consumption between individuals with hyperuricemia and controls in the 1:1 propensity score–matched study population.

Food group	Men			Women		
	Controls (*N* = 164)	Hyperuricemia (*N* = 164)	*p*-value	Controls (*N* = 226)	Hyperuricemia (*N* = 226)	*p*-value
Whole grains	7.1 ± 6.5	7.2 ± 6.6	0.451	7.8 ± 6.0	6.4 ± 5.7	**0.036**
Refined grains	13.5 ± 7.8	13.5 ± 8.2	0.666	8.4 ± 5.9	9.7 ± 7.0	**0.031**
Snacks	2.2 ± 2.6	2.4 ± 2.9	0.460	2.3 ± 3.5	2.4 ± 3.9	0.731
Fats and oils	0.3 ± 1.2	0.3 ± 1.1	0.921	0.3 ± 0.8	0.3 ± 0.9	0.832
Roots and tubers	1.2 ± 1.8	1.0 ± 1.1	0.295	1.6 ± 1.9	1.6 ± 1.9	0.562
Legumes soy products	3.6 ± 3.3	4.0 ± 3.8	0.402	3.7 ± 2.9	4.2 ± 4.2	0.072
Nuts and seeds	0.5 ± 1.0	0.4 ± 0.9	0.293	0.6 ± 1.4	0.6 ± 1.2	0.520
Vegetables	28.8 ± 15.1	29.8 ± 17.2	0.610	26.5 ± 16.1	26.9 ± 15.8	0.755
Mushrooms	0.6 ± 1.0	0.8 ± 2.6	0.474	0.6 ± 0.9	0.8 ± 1.3	0.127
Fruits	6.0 ± 5.9	5.9 ± 5.4	0.888	8.3 ± 6.7	9.1 ± 8.0	0.138
Red meat	4.5 ± 3.7	4.4 ± 3.3	0.390	2.7 ± 2.8	2.8 ± 2.5	0.629
White meat	1.0 ± 1.0	1.0 ± 0.9	0.796	0.8 ± 1.0	0.8 ± 0.8	0.612
Eggs	3.4 ± 3.4	3.3 ± 3.0	0.649	3.1 ± 3.1	3.6 ± 3.2	0.100
Fish	2.6 ± 2.9	2.9 ± 3.6	0.303	2.8 ± 2.9	3.3 ± 3.9	0.071
Other seafood and shellfish	1.3 ± 1.7	1.2 ± 1.6	0.201	0.8 ± 1.1	1.0 ± 1.4	**0.045**
Seaweeds	3.1 ± 2.8	3.1 ± 3.2	0.990	3.2 ± 2.9	3.7 ± 3.4	0.120
Milk and dairy products	8.4 ± 9.7	9.0 ± 8.3	0.721	5.9 ± 5.2	6.7 ± 8.9	0.228
Beverages	45.8 ± 125.7	35.4 ± 78.6	0.171	16.7 ± 39.4	23.8 ± 71.2	0.344
Soju (Korean distilled liquor)	1.8 ± 2.4	2.9 ± 4.4	**0.040**	0.3 ± 0.9	0.7 ± 1.6	**0.012**
Beer	3.6 ± 7.0	3.7 ± 6.9	0.674	1.2 ± 3.1	3.0 ± 11.6	**0.035**
Makgeolli (Korean rice wine)	0.5 ± 2.1	0.6 ± 1.5	0.935	0.1 ± 0.9	0.5 ± 5.9	0.365

Data are represented as mean ± standard deviation for continuous variables.

Age and BMI were matched in a 1:1 ratio using propensity score matching. *p*-values were calculated using generalized estimating equations with additional adjustments for alcohol consumption, smoking status, physical activity, and total energy intake. All *p*-values are two-sided. Statistical significance is defined as *p* < 0.05, and statistically significant results are highlighted in bold.

### 3.4 KHEI component score distributions in hyperuricemia and control groups

**Figure 2:**
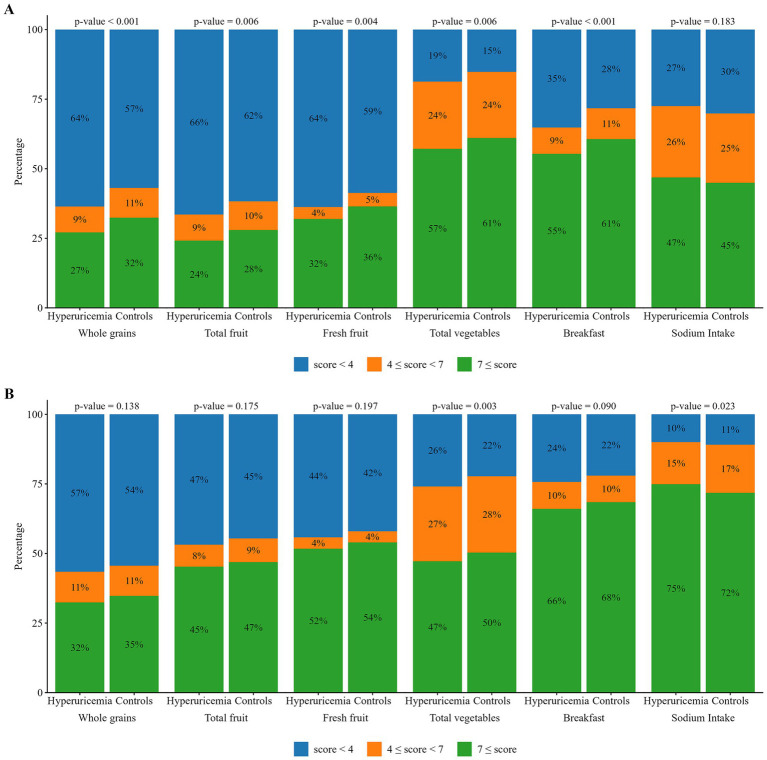
Distribution of participants across score categories for Korean Healthy Eating Index (KHEI) components showing significant differences, stratified by hyperuricemia status and sex. Scores were classified as low (<4), moderate (4–7), or high (≥7). Statistical comparisons were conducted using the chi-squared test. KHEI component scores should be interpreted according to the directionality of each component. For adequacy components (e.g., whole grains, fruits, vegetables, breakfast), higher scores indicate higher intake and better dietary quality; for moderation components (e.g., sodium intake), higher scores reflect lower intake and better dietary quality. **(A)** Male participants; **(B)** Female participants.

The distribution of the participants across the score categories for the KHEI components, revealing significant differences, is shown in Figure 2. In men, the proportion of individuals with higher component scores (≥7) for “whole grains,” “total fruit,” “fresh fruit,” “total vegetables,” and “breakfast” was significantly lower in the hyperuricemia group than in the control group (all *p* < 0.01; Figure 2A). In women, the proportion of participants with higher scores for “total vegetables” was significantly lower in the hyperuricemia group (*p* = 0.003), and the proportion of individuals with higher scores for “sodium intake” was significantly greater in the hyperuricemia group (*p* = 0.023; Figure 2B) than the control groups. No significant differences were observed among the other components.

### 3.5 Sensitivity analysis

In sensitivity analyses with additional adjustment for serum creatinine, several associations observed in women were attenuated and no longer statistically significant. Specifically, the between-group differences in overall HEI score (Table 1), zinc and sodium intake relative to the KDRIs (Table 2), and weekly intake of whole grains and other non-fish seafood and shellfish (Table 3) were no longer significant with additional adjustment. Importantly, the overall direction and pattern of associations remained largely consistent. Detailed results are provided in Supplementary Tables S2–S4.

## 4 Discussion

A large, nationally representative Korean cohort was analyzed, with 1:1 PSM to minimize confounding due to age and BMI. Individuals with hyperuricemia exhibited distinct and unfavorable dietary patterns compared with matched controls. These included lower intake of carbohydrates, fiber, and several key micronutrients, higher protein intake among women, and greater consumption of refined grains, shellfish, and alcohol. Collectively, these differences were associated with a poorer overall diet, suggesting the significant association of dietary composition and habits with hyperuricemia, which is beyond traditional metabolic factors.

### 4.1 Nutrient intake and hyperuricemia risk

Our DRI-based analysis demonstrated a lower proportion of individuals whose carbohydrate intake met or exceeded the DRI compared with controls in both sexes of the hyperuricemia group. Women with hyperuricemia had a higher proportion exceeding the protein DRI. This pattern suggests a relative shift in macronutrient distribution between the hyperuricemia and control groups. Low-carbohydrate diets (LCDs) have received considerable attention due to their proposed metabolic benefits based on the carbohydrate–insulin axis hypothesis (27, 28). However, LCDs generally promote increased consumption of protein and fat. A Japanese study also reported that the purine content of LCDs could exceed the recommended limits by approximately 1.7-fold (29). Mounting evidence suggests that the dietary sources of protein and fat (i.e., plant-based vs. animal-based) used to replace carbohydrates may be a critical factor influencing uric acid levels (30, 31).

Both men and women with hyperuricemia showed significantly lower intakes of dietary fiber, magnesium, and zinc, with additional deficiencies in folate and iron among men. Consistent with previous evidence, these nutrients were inversely associated with hyperuricemia. Dietary fiber may enhance satiety and promote intestinal motility, thereby facilitating uric acid excretion via the gastrointestinal tract (32, 33). Experimental studies in animal models (34) and a randomized crossover trial in patients with end-stage renal disease (35) suggest that certain fermentable fibers, such as inulin-type prebiotics, are associated with changes in serum uric acid levels, potentially through gut microbiota–mediated and inflammatory pathways, as well as through the modulation of intestinal uric acid excretion and/or reabsorption. Magnesium deficiency is associated with impaired renal urate elimination (36), whereas zinc and folate are involved in the regulation of oxidative stress and purine metabolism (37). Although the role of iron remains complex, animal-derived iron increases SUA, whereas plant-derived iron may be protective. Our findings underscore the importance of nutrient quality and quantity (38).

Vitamin B1, B2, and C intake was significantly reduced in men with hyperuricemia, which is consistent with findings of previous studies that reported inverse associations with SUA levels (39–41). Experimental and clinical studies have reported inverse associations between vitamins B1 and C and SUA levels, potentially through antioxidant mechanisms (39, 41). In particular, vitamin C has been consistently associated with modest reductions in SUA and gout risk (41).

Interestingly, a smaller proportion of individuals with hyperuricemia exceeded the sodium KDRI than the controls. While high sodium levels are a cardiovascular hazard, paradoxical associations between higher sodium intake and lower SUA levels have been reported, possibly due to effects on renal urate handling (42, 43). However, reverse causation cannot be excluded. Individuals with hyperuricemia had a higher prevalence of hypertension and may have already adopted a low-sodium diet following medical advice. Therefore, the lower sodium intake observed in the hyperuricemia group may reflect prior dietary modification, in addition to potential effects of sodium intake on uric acid metabolism. Similar behavior-related dietary changes have been reported in population-based surveys (44, 45). Future longitudinal studies are warranted to clarify the temporal relationships among sodium intake, hypertension, and serum uric acid levels.

Our results emphasize that hyperuricemia is linked not only to the consumption of purine-rich foods but also to broader deficiencies in micronutrients and overall diet quality.

### 4.2 Dietary habits and hyperuricemia risk

Women with hyperuricemia reported a greater consumption of refined grains and a lower intake of whole grains than the controls. Whole grains are rich in fiber, minerals, and phytoestrogens and have anti-inflammatory and glucose-regulating properties (46). Consumption of whole-grain and coarse-grain foods is inversely associated with hyperuricemia and gout, likely involving favorable modulation of the gut microbiota (47, 48).

In contrast, women with hyperuricemia consumed more shellfish and other non-fish seafood. Although nutrient-dense, these foods are high in purines, which are associated with higher SUA levels (4, 49, 50). A meta-analysis reported a 47% increased risk of hyperuricemia associated with seafood consumption, particularly among individuals with impaired renal urate excretion (49).

Alcohol intake also differed according to hyperuricemia status. Although beer, with its high purine content, is a well-established risk factor, a significant association was observed only in women (51). In contrast, soju consumption was higher in men and women with hyperuricemia. While the purine levels in soju are lower than those in beer, higher soju consumption is associated with elevated SUA levels in observational studies, potentially reflecting reduced renal urate clearance related to alcohol intake.

These results suggest that dietary habits related to staple foods and alcohol consumption are associated with hyperuricemia, indicating the potential benefits of reducing intake of refined grains, shellfish, and alcohol while promoting whole-grain intake.

### 4.3 Dietary quality and hyperuricemia risk

Dietary quality, as measured using the KHEI, was lower in individuals of both sexes with hyperuricemia. Lower scores were particularly evident among men for whole grains, fruits, vegetables, and breakfast consumption. In women, the difference was limited to total vegetable intake, with a greater proportion meeting the sodium-related recommendations. These findings suggest potential sex-specific differences in the dietary patterns related to hyperuricemia.

Vegetables and fruits are rich in polyphenols, vitamin C, fiber, and essential micronutrients with antioxidant and anti-inflammatory properties. Consistent with our results, previous studies have shown that low vegetable consumption (defined as intake fewer than three times per week) is associated with an approximately two-fold higher prevalence of hyperuricemia (52). Although fruits contain fructose and small amounts of purines, their beneficial compounds may partly explain the inverse associations observed with SUA levels (53). Polyphenols, in particular, may influence uric acid metabolism through inhibition of xanthine oxidase, reduced renal reabsorption, and enhanced urate excretion (54). The timing of meals may also influence the risk of hyperuricemia. Studies have shown that irregular breakfast consumption and higher energy intake during dinner are associated with an increased risk. Reallocating even 5% of dinner calories to breakfast was associated with a 13–20% reduction in hyperuricemia risk (55) underscoring the importance of both dietary composition and temporal distribution.

Overall, more dietary factors were associated with hyperuricemia in men than in women, likely reflecting differences in UA metabolism, including the protective effects of estrogen, as well as the generally higher KHEI scores among women. The convergence of our nutrient-based and diet quality analyses supports the hypothesis that plant-based dietary patterns are consistently associated with lower SUA levels, potentially through multiple mechanisms, including anti-inflammatory effects, reduced dietary acid load, and modulation of purine metabolism. Taken together, these findings emphasize that nutrient adequacy and dietary behaviors, such as regular breakfast consumption and moderate alcohol consumption, should be considered in the management of hyperuricemia.

This study has some limitations. First, owing to the observational and cross-sectional design, a causal relationship between dietary factors and hyperuricemia could not be established. Second, dietary data were obtained using a 24-h dietary recall and FFQ, both of which are prone to recall bias and measurement errors, particularly when quantifying nutrient intake. Third, residual confounding factors, such as genetic predisposition, medication use, and renal function, could not be excluded. Finally, nutrient intake was evaluated based on the 2020 KDRIs, which are suitable for the Korean population but may limit generalizability to other populations with differing dietary standards. Future longitudinal studies incorporating repeated dietary assessments are better suited to evaluate dose–response, threshold, or non-linear relationships between nutrient intake, dietary quality, and hyperuricemia risk.

Nevertheless, this study is valuable as it has leveraged a large, nationally representative Korean dataset and applied a rigorous 1:1 PSM to minimize confounding due to age and BMI, thereby ensuring robust and generalizable comparisons between individuals with and without hyperuricemia. This study offers a comprehensive and culturally relevant examination of dietary patterns linked to hyperuricemia, combining quantitative assessments of nutrient intake based on the KDRIs with qualitative evaluations of overall diet quality using the KHEI. Moreover, the inclusion of detailed sex-specific analysis highlights critical behavioral differences between men and women, adding depth and clinical relevance to the findings. Collectively, these methodological strengths position this study as a significant contribution to our understanding of modifiable dietary factors in the management and prevention of hyperuricemia.

## 5 Conclusion

This study highlights the importance of a comprehensive approach to hyperuricemia, encompassing nutrient intake, dietary habits, and overall dietary quality. These findings may inform the consideration of individualized dietary strategies that consider food composition and consumption patterns in relation to hyperuricemia. In this context, potential dietary approaches may emphasize: (i) balanced macronutrient intake; (ii) increased consumption of fiber-rich plant foods; (iii) sufficient intake of key micronutrients; (iv) inclusion of whole grains; and (v) regular consumption of breakfast. However, given the cross-sectional nature of this study, all findings should be interpreted as associations rather than causal relationships. Further longitudinal and interventional studies are warranted to establish causality and to support the development of evidence-based dietary guidelines for personalized hyperuricemia management.

## Data Availability

Publicly available datasets were analyzed in this study. This data can be found here: Korea National Health and Nutrition Examination Survey (KNHANES), conducted by the Korea Disease Control and Prevention Agency (KDCA). Data are available upon application and approval at: https://knhanes.kdca.go.kr.

## Footnotes

1
http://www.R-project.org/
