# Supplementary material for: Nutrient intake and diet quality in individuals with hyperuricemia: a matched population study
Source: Front Nutr. 2026 Feb 4;13:1719104. doi: 10.3389/fnut.2026.1719104 (PMC12913185; doi:10.3389/fnut.2026.1719104)
Supplement: Supplementary file 1 [file Table_1.docx]

**Supplementary Table S1.** Sex- and age-specific dietary reference intakes based on the 2020 Korean Dietary Reference Intakes (KDRIs)

|  | Men | | | | |  | Women | | | | |
| --- | --- | --- | --- | --- | --- | --- | --- | --- | --- | --- | --- |
|  | 19-29 | 30-49 | 50-64 | 65-74 | ≥75 |  | 19-29 | 30-49 | 50-64 | 65-74 | ≥75 |
| Fiber, g/day | 30 | 30 | 30 | 25 | 25 |  | 20 | 20 | 20 | 20 | 20 |
| Mg, mg/day | 360 | 370 | 370 | 370 | 370 |  | 280 | 280 | 280 | 280 | 280 |
| Ca, mg/day | 800 | 800 | 750 | 700 | 700 |  | 700 | 700 | 800 | 800 | 800 |
| Iron, mg/day | 10 | 10 | 10 | 9 | 9 |  | 14 | 14 | 8 | 8 | 7 |
| Na, mg/day | 1500 | 1500 | 1500 | 1300 | 1100 |  | 1500 | 1500 | 1500 | 1300 | 1100 |
| K, mg/day | 3500 | 3500 | 3500 | 3500 | 3500 |  | 3500 | 3500 | 3500 | 3500 | 3500 |
| Zinc, mg/day | 10 | 10 | 10 | 9 | 9 |  | 8 | 8 | 8 | 7 | 7 |
| Vitamin A, μg RAE | 800 | 800 | 750 | 700 | 700 |  | 650 | 650 | 600 | 600 | 600 |
| Vitamin E, mg/day | 12 | 12 | 12 | 12 | 12 |  | 12 | 12 | 12 | 12 | 12 |
| Vitamin B1, mg/day | 1.2 | 1.2 | 1.2 | 1.1 | 1.1 |  | 1.1 | 1.1 | 1.1 | 1.0 | 0.8 |
| Vitamin B2, mg/day | 1.5 | 1.5 | 1.5 | 1.4 | 1.3 |  | 1.2 | 1.2 | 1.2 | 1.1 | 1.0 |
| Niacin, mg/day | 16 | 16 | 16 | 14 | 13 |  | 14 | 14 | 14 | 13 | 12 |
| Vitamin C, mg/day | 100 | 100 | 100 | 100 | 100 |  | 100 | 100 | 100 | 100 | 100 |
| Folate, μg/day | 400 | 400 | 400 | 400 | 400 |  | 400 | 400 | 400 | 400 | 400 |

Age groups were defined as 19–29, 30–49, 50–64, 65–74, and ≥75 years according to the 2020 KDRIs.

Mg, magnesium; Ca, calcium; Na, sodium; K, potassium; RAE, retinol activity equivalent. Reference values for nutrients were based on the following criteria according to sex and age:

Recommended nutrient intake (RNI): Mg, Ca, iron, vitamins A, B1, B2, and C, niacin, zinc, and folate.

Adequate intake (AI): fiber, Na, K, and vitamin E

**Supplementary Table S2.** Characteristics of the 1:1 propensity score-matched study population with additional adjustments for serum creatinine

|  | Male | | |  | Female | | |
| --- | --- | --- | --- | --- | --- | --- | --- |
|  | Controls  (N = 2,048) | Hyperuricemia  (N = 2,048) | p-value |  | Controls  (N = 3,086) | Hyperuricemia  (N = 3,086) | p-value |
| Age (y) | 48.9 ± 17.0 | 48.4 ± 17.3 | - |  | 54.9 ± 15.4 | 54.5 ± 17.4 | - |
| BMI (kg/m^2^) | 25.8 ± 3.7 | 25.7 ± 3.5 | - |  | 25.1 ± 3.9 | 25.1 ± 3.9 | - |
| WC (cm) | 90.1 ± 9.7 | 90.3 ± 9.1 | **<0.001** |  | 84.0 ± 10.2 | 84.4 ± 10.1 | **<0.001** |
| MBP (mmHg) | 92.5 ± 10.0 | 93.9 ± 11.0 | **<0.001** |  | 89.8 ± 11.0 | 90.3 ± 11.1 | **0.028** |
| Alcohol intake (yes) | 1,421 (69.4%) | 1,491 (72.8%) | **<0.001** |  | 1,140 (36.9%) | 1,199 (38.9%) | 0.119 |
| Smoking (yes) | 621 (30.3%) | 654 (31.9%) | 0.430 |  | 122 (4.0%) | 192 (6.2%) | **<0.001** |
| Physical activity (yes) | 1,011 (49.4%) | 973 (47.5%) | 0.187 |  | 1,179 (38.2%) | 1,189 (38.5%) | 0.805 |
| Hypertension (yes) | 727 (35.5%) | 816 (39.8%) | **0.007** |  | 1,125 (36.5%) | 1,293 (41.9%) | **<0.001** |
| Diabetes (yes) | 367 (17.9%) | 252 (12.3%) | **<0.001** |  | 490 (15.9%) | 541 (17.5%) | 0.483 |
| Dyslipidemia (yes) | 759 (37.1%) | 940 (45.9%) | **<0.001** |  | 1,189 (38.5%) | 1,349 (43.7%) | **<0.001** |
| Metabolic syndrome (yes) | 707 (34.5%) | 851 (41.6%) | **<0.001** |  | 1,065 (34.5%) | 1,371 (44.4%) | **<0.001** |
| Glucose (mg/dL) | 105.5 ± 27.5 | 101.9 ± 19.3 | **<0.001** |  | 101.7 ± 23.6 | 102.6 ± 22.8 | 0.408 |
| TC (mg/dL) | 189.8 ± 38.8 | 198.2 ± 40.3 | **<0.001** |  | 193.4 ± 39.8 | 197.1 ± 41.8 | **<0.001** |
| TG (mg/dL) | 149.0 ± 112.0 | 191.7 ± 159.6 | **<0.001** |  | 115.1 ± 69.6 | 139.3 ± 96.0 | **<0.001** |
| HDL-C (mg/dL) | 47.7 ± 11.3 | 45.0 ± 10.3 | **<0.001** |  | 53.9 ± 12.4 | 51.5 ± 12.4 | **<0.001** |
| LDL-C (mg/dL) | 114.6 ± 34.2 | 119.5 ± 35.9 | **<0.001** |  | 116.9 ± 35.3 | 119.0 ± 37.5 | **0.003** |
| Uric acid (mg/dL) | 5.5 ± 0.9 | 7.8 ± 0.8 | **<0.001** |  | 4.1 ± 0.7 | 5.9 ± 0.8 | **<0.001** |
| Creatinine | 0.9 ± 0.2 | 1.0 ± 0.3 | **<0.001** |  | 0.7 ± 0.2 | 0.8 ± 0.3 | **<0.001** |
| HEI | 60.6 ± 13.6 | 58.3 ± 13.2 | **<0.001** |  | 63.9 ± 13.1 | 62.8 ± 13.6 | 0.093 |
| Total energy intake, kcal/day | 2,277.6 ± 969.2 | 2,238.2 ± 952.9 | 0.262 |  | 1,604.8 ± 655.4 | 1,569.1 ± 666.2 | 0.143 |
| Carbohydrate (%) | 59.1 ± 14.3 | 57.4 ± 15.1 | **<0.001** |  | 65.2 ± 12.6 | 63.7 ± 13.7 | **<0.001** |
| Protein (%) | 14.9 ± 4.4 | 14.7 ± 4.8 | 0.117 |  | 14.3 ± 4.1 | 14.6 ± 4.5 | **0.010** |
| Fat (%) | 20.5 ± 9.4 | 20.7 ± 9.9 | 0.381 |  | 19.2 ± 9.4 | 19.8 ± 9.9 | **0.006** |
| SFA (%) | 6.4 ± 3.6 | 6.6 ± 3.7 | 0.184 |  | 6.0 ± 3.6 | 6.3 ± 3.9 | **0.004** |
| MUFA (%) | 6.6 ± 3.8 | 6.7 ± 4.0 | 0.164 |  | 6.0 ± 3.7 | 6.2 ± 3.8 | **0.046** |
| PUFA (%) | 5.4 ± 2.9 | 5.3 ± 3.0 | 0.274 |  | 5.3 ± 2.8 | 5.3 ± 3.0 | 0.363 |
| PUFA / SFA | 1.1 ± 0.7 | 1.0 ± 0.6 | **0.017** |  | 1.1 ± 0.7 | 1.1 ± 0.8 | 0.155 |
| PUFA + MUFA /SFA | 15.2 ± 10.6 | 14.6 ± 10.2 | 0.130 |  | 10.7 ± 7.4 | 10.6 ± 7.7 | 0.250 |
| Omega-3 fatty acid, g/day | 2.1 ± 2.0 | 2.0 ± 1.9 | 0.211 |  | 1.7 ± 2.2 | 1.63 ± 1.84 | 0.855 |
| Omega-6 fatty acid, g/day | 11.9 ± 9.2 | 11.5 ± 8.8 | 0.160 |  | 8.0 ± 6.3 | 8.0 ± 6.5 | 0.202 |
| Cholesterol, mg/day | 291.6 ± 250.8 | 279.5 ± 240.3 | 0.146 |  | 188.7 ± 171.5 | 192.5 ± 188.5 | **0.047** |
| Fiber, g/day | 28.3 ± 14.5 | 26.4 ± 13.8 | **0.002** |  | 24.5 ± 13.6 | 23.2 ± 13.4 | **0.021** |
| Mg, mg/day | 358.5 ± 160.8 | 335.7 ± 151.9 | **<0.001** |  | 284.1 ± 142.5 | 270.9 ± 134.0 | **0.018** |
| Ca, mg/day | 558.7 ± 307.9 | 522.6 ± 293.6 | **0.002** |  | 455.3 ± 296.2 | 437.5 ± 274.2 | 0.206 |
| Iron, mg/day | 12.7 ± 8.3 | 11.9 ± 7.5 | **0.003** |  | 9.6 ± 5.7 | 9.1 ± 5.7 | 0.119 |
| Na, mg/day | 3,998.6 ± 2,184.2 | 3,857.4 ± 2,178.8 | 0.175 |  | 2,806.6 ± 1,885.4 | 2,644.0 ± 1,675.5 | **0.004** |
| K, mg/day | 3,123.9 ± 1,395.7 | 2,962.7 ± 1,351.3 | **0.002** |  | 2,558.1 ± 1,296.8 | 2,459.1 ± 1,274.7 | 0.164 |
| Zinc, mg/day | 12.1 ± 6.2 | 11.6 ± 6.0 | **0.039** |  | 8.8 ± 4.2 | 8.6 ± 4.3 | 0.916 |
| Vitamin A, μg RAE | 425.1 ± 538.9 | 402.3 ± 393.4 | 0.313 |  | 350.8 ± 364.8 | 342.9 ± 375.9 | 0.833 |
| Vitamin E, mg/day | 7.8 ± ±4.6 | 7.5 ± 4.4 | **0.031** |  | 5.8 ± 3.5 | 5.6 ± 3.6 | 0.960 |
| Vitamin B1, mg/day | 1.5 ± 0.9 | 1.4 ± 0.9 | 0.182 |  | 1.1 ± 0.6 | 1.0 ± 0.6 | 0.140 |
| Vitamin B2, mg/day | 1.82 ± 1.04 | 1.8 ± 1.0 | 0.109 |  | 1.3 ± 0.7 | 1.3 ± 0.8 | 0.901 |
| Niacin, mg/day | 15.3 ± 9.2 | 14.9 ± 9.1 | 0.490 |  | 10.6 ± 5.9 | 10.7 ± 6.5 | 0.050 |
| Vitamin C, mg/day | 69.9 ± 107.1 | 64.2 ± 106.7 | 0.203 |  | 62.8 ± 72.9 | 59.5 ± 72.6 | 0.430 |
| Folate, μg/day | 361.4 ± 183.3 | 330.6 ± 165.6 | **<0.001** |  | 298.3 ± 154.8 | 283.8 ± 156.5 | **0.036** |

BMI, body mass index; WC, waist circumference; MBP, mean blood pressure; TC, total cholesterol; TG, triglycerides; HDL-C, high-density lipoprotein cholesterol; LDL-C, low-density lipoprotein cholesterol; HEI, healthy eating index; SFA, saturated fatty acids; MUFA, monounsaturated fatty acids; PUFA, polyunsaturated fatty acids; Mg, magnesium; Ca, calcium; Na, sodium; K, potassium; RAE, retinol activity equivalents.

Data are presented as mean ± standard deviation for continuous variables or as number (%) for categorical variables.

Age and BMI were matched in a 1:1 ratio using propensity score matching. p-values were calculated using generalized estimating equations with additional adjustments for alcohol consumption, smoking status, physical activity, and total energy intake. This table presents results from sensitivity analyses with further adjustment for serum creatinine. All p-values are two-sided. Statistical significance is defined as p < 0.05, and statistically significant results are highlighted in bold.

**Supplementary Table S3.** Comparison of daily nutrient intake between individuals with hyperuricemia and controls in the 1:1 propensity score-matched population with additional adjustments for serum creatinine

| Nutrients | Male | | | | |  | Female | | | | |
| --- | --- | --- | --- | --- | --- | --- | --- | --- | --- | --- | --- |
|  | Controls (N = 2,048) | | Hyperuricemia (N = 2,048) | | p-value |  | Controls (N = 3,086) | | Hyperuricemia (N = 3,086) | | p-value |
|  | Above DRI group, n (%) | Below DRI group, n (%) | Above DRI group, n (%) | Below DRI group, n(%) |  |  | Above DRI group, n (%) | Below DRI group, n (%) | Above DRI group, n (%) | Below DRI group, n(%) |  |
| Carbohydrate (%) | 801 (39.1%) | 1,247 (60.9%) | 696 (34.0%) | 1,352 (66.0%) | **<0.001** |  | 1,667 (54.0%) | 1,419 (46.0%) | 1,545 (50.1%) | 1,541 (49.9%) | **<0.001** |
| Protein (%) | 226 (11.0%) | 1,822 (89.0%) | 220 (10.7%) | 1,828 (89.3%) | 0.485 |  | 251 (8.1%) | 2,835 (91.9%) | 327 (10.6%) | 2,759 (89.4%) | **0.001** |
| Fat (%) | 301 (14.7%) | 1,747 (85.3%) | 343 (16.7%) | 1,705 (83.3%) | 0.054 |  | 414 (13.4%) | 2,672 (86.6%) | 463 (15.0%) | 2,623 (85.0%) | 0.170 |
| Cholesterol, mg/day | 788 (38.5%) | 1,260 (61.5%) | 752 (36.7%) | 1,296 (63.3%) | 0.209 |  | 686 (22.2%) | 2,400 (77.8%) | 688 (22.3%) | 2,398 (77.7%) | 0.623 |
| Fiber, g/day^#^ | 838 (40.9%) | 1,210 (59.1%) | 735 (35.9%) | 1,313 (64.1%) | **0.037** |  | 1,750 (56.7%) | 1,336 (43.3%) | 1,585 (51.4%) | 1,501 (48.6%) | **0.036** |
| Mg, mg/day | 831 (40.6%) | 1,217 (59.4%) | 719 (35.1%) | 1,329 (64.9%) | **0.002** |  | 1,387 (44.9%) | 1,699 (55.1%) | 1,233 (40.0%) | 1,853 (60.0%) | **0.011** |
| Ca, mg/day | 408 (19.9%) | 1,640 (80.1%) | 327 (16.0%) | 1,721 (84.0%) | **0.009** |  | 340 (11.0%) | 2,746 (89.0%) | 329 (10.7%) | 2,757 (89.3%) | 0.975 |
| Iron, mg/day | 1,218 (59.5%) | 830 (40.5%) | 1,100 (53.7%) | 948 (46.3%) | **0.001** |  | 1,252 (40.6%) | 1,834 (59.4%) | 1,185 (38.4%) | 1,901 (61.6%) | 0.803 |
| Na, mg/day | 1,922 (93.8%) | 126 (6.2%) | 1,874 (91.5%) | 174 (8.5%) | **0.038** |  | 2,509 (81.3%) | 577 (18.7%) | 2,445 (79.2%) | 641 (20.8%) | 0.474 |
| K, mg/day | 689 (33.6%) | 1,359 (66.4%) | 609 (29.7%) | 1,439 (70.3%) | **0.040** |  | 570 (18.5%) | 2,516 (81.5%) | 534 (17.3%) | 2,552 (82.7%) | 0.943 |
| Zinc, mg/day | 1,240 (60.5%) | 808 (39.5%) | 1,148 (56.1%) | 900 (43.9%) | **0.024** |  | 1,736 (56.3%) | 1,350 (43.7%) | 1,594 (51.7%) | 1,492 (48.3%) | 0.067 |
| Vitamin A, μg RAE | 190 (9.3%) | 1,858 (90.7%) | 207 (10.1%) | 1,841 (89.9%) | 0.117 |  | 381 (12.3%) | 2,705 (87.7%) | 375 (12.2%) | 2,711 (87.8%) | 0.549 |
| Vitamin E, mg/day | 271 (13.2%) | 1,777 (86.8%) | 253 (12.4%) | 1,795 (87.6%) | 0.802 |  | 157 (5.1%) | 2,929 (94.9%) | 158 (5.1%) | 2,928 (94.9%) | 0.762 |
| Vitamin B1, mg/day | 1,152 (56.3%) | 896 (43.8%) | 1,071 (52.3%) | 977 (47.7%) | **0.038** |  | 1,286 (41.7%) | 1,800 (58.3%) | 1,228 (39.8%) | 1,858 (60.2%) | 0.688 |
| Vitamin B2, mg/day | 1,191 (58.2%) | 857 (41.8%) | 1,113 (54.3%) | 935 (45.7%) | **0.047** |  | 1,621 (52.5%) | 1,465 (47.5%) | 1,537 (49.8%) | 1,549 (50.2%) | 0.669 |
| Niacin, mg/day | 785 (38.3%) | 1,263 (61.7%) | 732 (35.7%) | 1,316 (64.3%) | 0.096 |  | 690 (22.4%) | 2,396 (77.6%) | 721 (23.4%) | 2,365 (76.6%) | 0.101 |
| Vitamin C, mg/day | 360 (17.6%) | 1,688 (82.4%) | 302 (14.7%) | 1,746 (85.3%) | **0.049** |  | 498 (16.1%) | 2,588 (83.9%) | 501 (16.2%) | 2,585 (83.8%) | 0.392 |
| Folate, μg/day | 705 (34.4%) | 1,343 (65.6%) | 591 (28.9%) | 1,457 (71.1%) | **0.003** |  | 630 (20.4%) | 2,456 (79.6%) | 600 (19.4%) | 2,486 (80.6%) | 0.592 |

DRI, dietary reference intake; Mg, magnesium; Ca, calcium; Na, sodium; K, potassium; RAE, retinol activity equivalent. Reference values for nutrients were based on the following criteria, according to sex and age:

Acceptable macronutrient distribution ranges (AMDR): carbohydrate (%), protein (%), and fat (%).

Recommended nutrient intake (RNI): Mg, Ca, iron, vitamins A, B1, B2, and C, niacin, zinc, and folate.

Adequate intake (AI): fiber, Na, K, and vitamin E

Cholesterol levels were set based on chronic disease endpoints.

Age and BMI were matched in a 1:1 ratio using propensity score matching. p-values were calculated using generalized estimating equations with additional adjustments for alcohol consumption, smoking status, physical activity, and total energy intake. This table presents results from sensitivity analyses with further adjustments for serum creatinine. All p-values are two-sided. Statistical significance is defined as p < 0.05, and statistically significant results are highlighted in bold.

**Supplementary Table S4.** Comparison of weekly dietary intake and alcohol consumption between individuals with hyperuricemia and controls in the 1:1 propensity score-matched population with additional adjustments for serum creatinine

|  | Male | | |  | Female4 | | |
| --- | --- | --- | --- | --- | --- | --- | --- |
|  | Controls  (N = 164) | Hyperuricemia  (N = 164) | p-value |  | Controls  (N = 226) | Hyperuricemia  (N = 226) | p-value |
| Whole grains | 7.1 ± 6.5 | 7.2 ± 6.6 | 0.833 |  | 7.8 ± 6.0 | 6.4 ± 5.7 | 0.090 |
| Refined grains | 13.5 ± 7.8 | 13.5 ± 8.2 | 0.973 |  | 8.4 ± 5.9 | 9.7 ± 7.0 | **0.044** |
| Snacks | 2.2 ± 2.6 | 2.4 ± 2.9 | 0.449 |  | 2.3 ± 3.5 | 2.4 ± 3.9 | 0.683 |
| Fats and oils | 0.3 ± 1.2 | 0.3 ± 1.1 | 0.662 |  | 0.3 ± 0.8 | 0.3 ± 0.9 | 0.893 |
| Roots and tubers | 1.2 ± 1.8 | 1.0 ± 1.1 | 0.353 |  | 1.6 ± 1.9 | 1.6 ± 1.9 | 0.591 |
| Legumes soy products | 3.6 ± 3.3 | 4.0 ± 3.8 | 0.308 |  | 3.7 ± 2.9 | 4.2 ± 4.2 | 0.056 |
| Nuts and seeds | 0.5 ± 1.0 | 0.4 ± 0.9 | 0.284 |  | 0.6 ± 1.4 | 0.6 ± 1.2 | 0.639 |
| Vegetables | 28.8 ± 15.1 | 29.8 ± 17.2 | 0.689 |  | 26.5 ± 16.1 | 26.9 ± 15.8 | 0.953 |
| Mushrooms | 0.6 ± 1.0 | 0.8 ± 2.6 | 0.472 |  | 0.6 ± 0.9 | 0.8 ± 1.3 | 0.123 |
| Fruits | 6.0 ± 5.9 | 5.9 ± 5.4 | 0.563 |  | 8.3 ± 6.7 | 9.1 ± 8.0 | 0.145 |
| Red meat | 4.5 ± 3.7 | 4.4 ± 3.3 | 0.345 |  | 2.7 ± 2.8 | 2.8 ± 2.5 | 0.689 |
| White meat | 1.0 ± 1.0 | 1.0 ± 0.9 | 0.976 |  | 0.8 ± 1.0 | 0.8 ± 0.8 | 0.550 |
| Eggs | 3.4 ± 3.4 | 3.3 ± 3.0 | 0.803 |  | 3.1 ± 3.1 | 3.6 ± 3.2 | 0.082 |
| Fish | 2.6 ± 2.9 | 2.9 ± 3.6 | 0.116 |  | 2.8 ± 2.9 | 3.3 ± 3.9 | 0.075 |
| Other seafood and shellfish | 1.3 ± 1.7 | 1.2 ± 1.6 | 0.409 |  | 0.8 ± 1.1 | 1.0 ± 1.4 | 0.057 |
| Seaweeds | 3.1 ± 2.8 | 3.1 ± 3.2 | 0.495 |  | 3.2 ± 2.9 | 3.7 ± 3.4 | 0.225 |
| Milk and dairy products | 8.4 ± 9.7 | 9.0 ± 8.3 | 0.555 |  | 5.9 ± 5.2 | 6.7 ± 8.9 | 0.244 |
| Beverages | 45.8 ± 125.7 | 35.4 ± 78.6 | 0.281 |  | 16.7 ± 39.4 | 23.8 ± 71.2 | 0.384 |
| Soju (Korean distilled liquor) | 1.8 ± 2.4 | 2.9 ± 4.4 | **0.014** |  | 0.3 ± 0.9 | 0.7 ± 1.6 | **0.008** |
| Beer | 3.6 ± 7.0 | 3.7 ± 6.9 | 0.674 |  | 1.2 ± 3.1 | 3.0 ± 11.6 | **0.045** |
| Makgeolli (Korean rice wine) | 0.5 ±2.1 | 0.6 ±1.5 | 0.506 |  | 0.1 ± 0.9 | 0.5 ± 5.9 | 0.397 |

Data are represented as mean ± standard deviation for continuous variables.

Age and BMI were matched in a 1:1 ratio using propensity score matching. p-values were calculated using generalized estimating equations with additional adjustments for alcohol consumption, smoking status, physical activity, and total energy intake. This table presents results from sensitivity analyses with further adjustments for serum creatinine. All p-values are two-sided. Statistical significance is defined as p < 0.05, and statistically significant results are highlighted in bold.
